# Integration of Thermo-Responsive Materials Applied to Bio-Inspired Structures

**DOI:** 10.3390/biomimetics10020068

**Published:** 2025-01-22

**Authors:** Elton Lima, Hilma Ferreira, Luís Mateus, Amilton Arruda

**Affiliations:** 1 CIAUD, Research Centre for Architecture, Urbanism and Design, Lisbon School of Architecture, Universidade de Lisboa, 1349-063 Lisboa, Portugal; lmmateus@fa.ulisboa.pt; 2 Biodesign Lab, Federal University of Pernambuco, Recife 50740-550, Brazil; hilma.santos@ufpe.br (H.F.); amilton.arruda@ufpe.br (A.A.)

**Keywords:** biomimicry, problem-based approach, crystallography, thermochromism, hydrogel, architecture, glass, film

## Abstract

This paper investigates the integration of thermo-responsive materials into bio-inspired structures, combining biomimicry and adaptive technologies in architecture. A problem-based biomimetic approach and a morphological analogy with the plate-type snowflake—known for its lightness, transparency, and crystalline organisation—were adopted to develop the geometry of an architectural pavilion. This research highlights glass as a main constructive material, analysing the potential of thermochromic film and the hydrogel technique, both inserted in the context of thermo-responsiveness. In this regard, the focus is on adaptations to temperature changes, exploring how these materials can alter their properties in response to solar incidence, offering solutions for energy efficiency, thermal regulation, and environmental adaptation. The pavilion demonstrates that this integration is feasible, and this is supported by an interdisciplinary approach that combines materials science, bio-inspired design, and practical experimentation. It also highlights biomimicry’s fundamental role as a tool for guiding the development of innovative architectural geometries, while thermo-responsive materials expand the possibilities for creating structures that are adaptable to temperature variations and solar exposure. The conclusion points to the applicability and relevance of this combination, highlighting the transformative potential of thermo-responsive materials in architectural projects, especially in the development of lightweight, transparent, and environmentally responsive structures.

## 1. Introduction

### 1.1. Biomimicry

Biomimicry is an interdisciplinary field that seeks solutions to human challenges by studying and emulating strategies found in nature. This approach combines biology with various other fields of knowledge, such as architecture, design, engineering, among others, in order to develop sustainable and efficient solutions.

The word biomimicry derives from the Greek *bios* (life) and *mimesis* (imitation), emphasising the central concept of learning from natural systems. The term emerged in 1982 but only became widespread in 1997 with Benyus’ work *Biomimicry: Innovation Inspired by Nature*, in which she proposes looking at nature as a model, measure, and mentor [1,2,3]. This perspective highlights nature as a source of knowledge, rather than merely an object of study, reinforcing the concept of “biomimetic epistemology”, explored by Dicks [3], which advocates for direct learning from natural processes to inspire sustainable innovations.

In this context, following these principles of Benyus that connect the concept of biomimicry with its object of study (nature), by looking at nature as a model, one studies organisms and ecosystems to understand how they solve complex problems; furthermore, one uses nature as a measure by evaluating the sustainability of human solutions based on natural patterns and processes and nature as a mentor by promoting a new way of thinking where natural systems are seen as inspiration rather than resources to be exploited.

Recent studies have emphasised the importance of categorising biomimetic approaches into levels such as form, which encompasses the surface properties of the organism; behaviour, related to the principles governing its actions; and ecosystem, which explores how the organism interacts with its environment—each contributing to a sustainable design [4]. For instance, in architecture, biomimicry applied at the ecosystem level has inspired circular resource cycles and energy-efficient systems, as demonstrated in several case studies reviewed by Verbrugghe et al. [5], such as the Eden Project in Cornwall, UK, and the Arab World Institute in Paris, France.

With all the infinite possibilities for applying and transposing natural knowledge into human solutions, whether products or processes, there is no single or well-defined biomimetic design method. Each project is unique and must address the specific needs of each field of knowledge, following technical and safety standards.

So, however many methods are discussed, biomimicry is essentially an association with some natural element that objectively or subjectively helps to develop practical solutions to some human challenge. Therefore, the process of emulation is open to different interpretations depending on the researcher and their needs.

In this context, among the various methods discussed in the literature, we can see the relevance of the most different analogies such as personal, direct, symbolic, fantastic [6], organic, classificatory, anatomical, ecological, Darwinian [7], morphological [8], sensory [9], functional, semantic [10], structural morphological, movement, and behavioural [11]. These various analogies reflect the interdisciplinary potential of biomimicry to address complex problems in innovative ways, as discussed by Sá and Viana [12].

**Figure 1:**
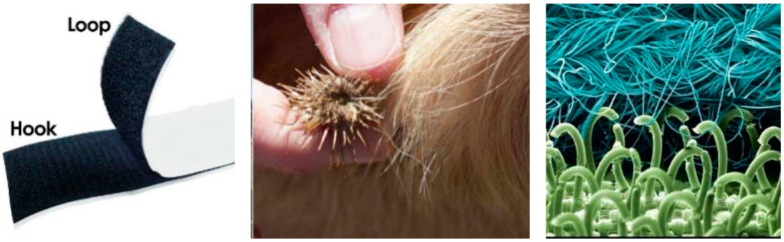
Velcro (**left**); natural inspiring element (**centre**); microscopic image of Velcro (**right**) [13].

For the purposes of this research, this paper focuses on the study of morphological analogy [8]. This involves analysing formal and structural attributes of nature to inspire the development of projects, products, and systems. A classic example is Velcro^®^ (Figure 1), created from the observation of plants of the Asteraceae family, whose microscopic, hooked structures served as a model for this fastening system.

These morphological analogies enhance creativity in design while enabling solutions aligned with sustainability goals, further reinforcing the transformative potential of biomimicry in architecture and engineering [5,12].

However, although the literature already presents examples of biomimetic applications at different architectural scales—such as structural systems based on form (e.g., the Eastgate Centre in Harare, designed by Mick Pearce, inspired by termite mounds to develop a ventilation system [5]) and sustainable buildings inspired by ecosystems (e.g., Council House 2 in Melbourne, designed by Mick Pearce and Rob Adams, drawing on principles observed in trees, such as air filtration and optimised energy flows [5])—there remains a need to expand studies that systematically integrate strategies inspired by natural elements with the use of adaptive materials, demonstrating clear benefits in terms of energy performance and sustainability.

This context has motivated the adoption of an approach combining inspiration from the shapes of snowflakes with thermally responsive materials, aiming to propose more efficient and environmentally responsive architectural solutions.

### 1.2. Snowflakes and the Study of Crystallography

The first scientific reference to snowflakes dates back to 1611 when Johannes Kepler published the treatise *On the Six-Cornered Snowflake*; he raised the question of why snowflakes have a sixfold symmetry—not referring to the atomistic point of view—and speculated that the hexagonal packing of spheres might have something to do with the shape of snow crystals [14,15,16,17]. Another historical reference dates to 1665, when Robert Hooke published the work *Micrographia*, containing sketches of elements that he was able to analyse through a microscope, which at the time was a recent invention; this volume contained the first graphic representations of snow crystals, revealing their complexity and structural symmetry [18]. Although questions and curiosities had been raised for centuries, many answers were only clarified later, when modern crystallography emerged in 1911, and snowflakes became objects of study.

Snowflakes begin to form when the air temperature is below 0 °C, so the water vapour in the atmosphere condenses and freezes, forming ice crystals [19]. As water vapour begins to condense in a cloud, the ice particle develops facets, turning into a small hexagonal prism, and as it grows, it remains in this simple faceted shape. When it gets bigger, branches begin to sprout from the six corners of the hexagon, which grow at roughly the same rate, since the atmospheric conditions of temperature and humidity are practically constant in the crystal as a whole. In the process of formation, the crystal is blown from side to side within the cloud, causing it to face random temperature changes, affecting its growth and shape, which nevertheless results in a complex branched structure [20]. As each crystal travels a different path within the cloud, and consequently faces different physical conditions, they also tend to be different in shape [21,22].

With this entire process of formation, it is very difficult—not impossible—for two snowflakes to have the same shape. This is because despite being composed of the same hydrogen and oxygen molecules, environmental conditions determine their unique crystalline structures. Crystals can therefore be classified into around 39 categories of solid precipitation, but scientists estimate that the possibilities for individual shapes are practically infinite. It is estimated that the number of possible shapes is much greater than the number of atoms in the universe [19,23]. By analogy, this great diversity of snowflake shapes would be as if each snowflake had its own DNA. However, scientists are still able to manage to collect and catalogue minute details of the morphological structures of snow crystals.

**Figure 2:**
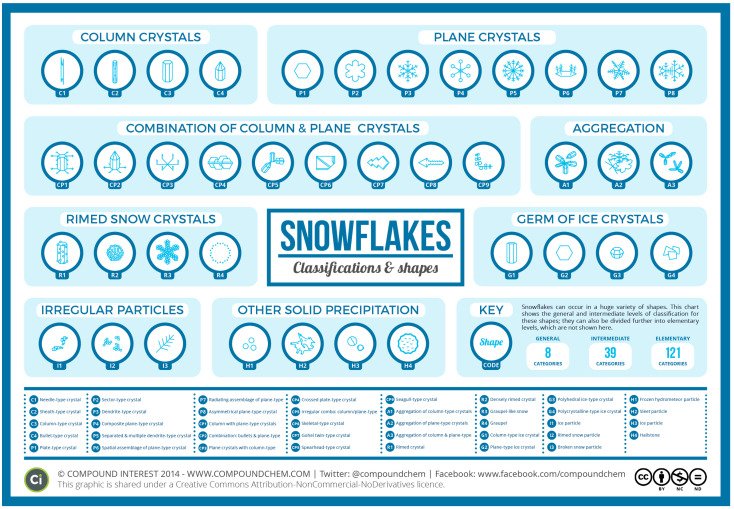
Graph created by British chemistry professor Andy Brunning, illustrating 39 types of solid precipitation, including 35 types of crystals or snowflakes, as well as hail and ice [24,25].

Over time, classifications have come up with different numbers for the total categories of solid precipitation. Figure 2 below is based on the work of Japanese researchers in which the 39 categories can be divided into 121 subcategories that can be separated into 8 broader groups [24,25,26].

**Figure 3:**
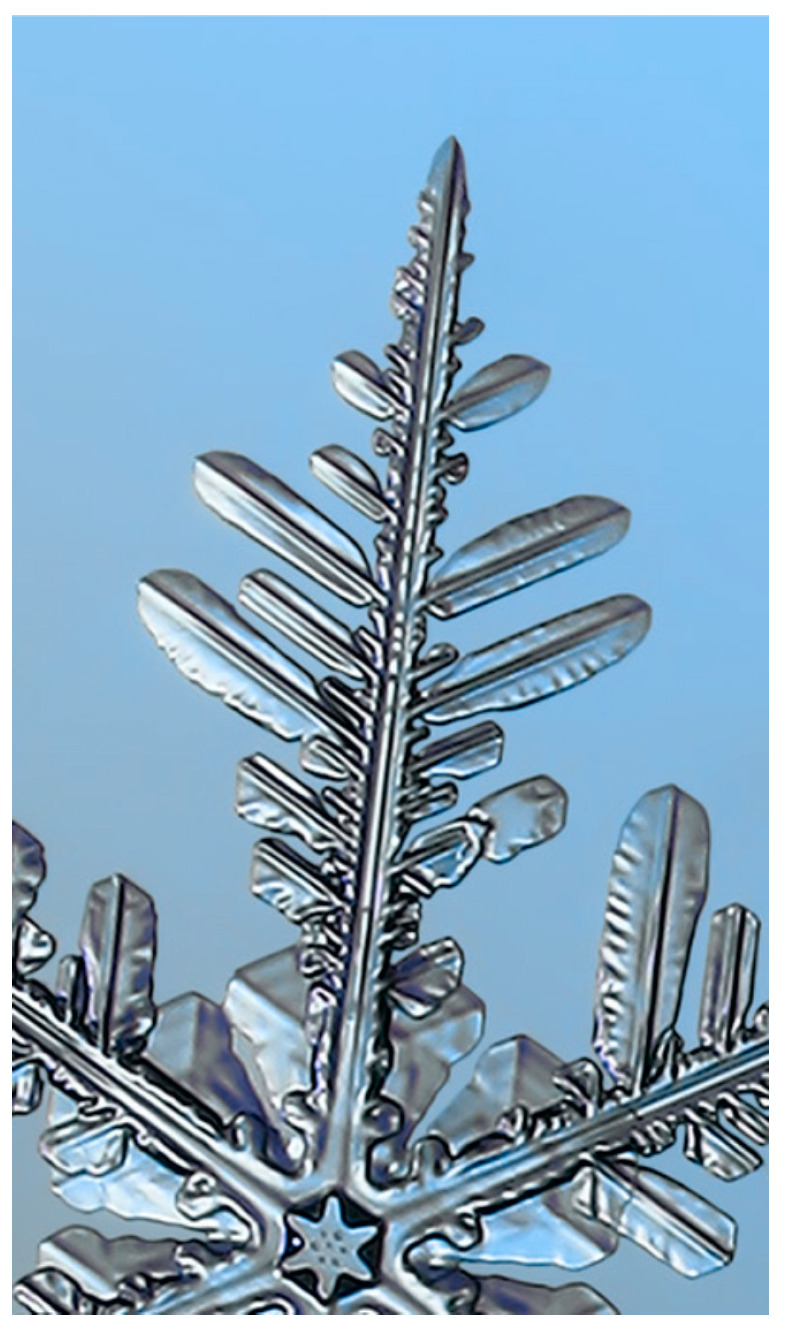
Ice dendrites [28].

The complexity of snowflake shapes is evidenced by their lacy and branched structure, which is made up of the way water molecules travel through the air to condense into a snow crystal; the more the molecules need to diffuse through the air to reach the crystal, the more the growth is slowed down [27]. In addition, the more water molecules that collide with the forming crystal, the faster it grows. This is called branching instability, which is responsible for the development of complex shapes, which are the small protrusions that develop on larger branches, thus forming side branches. When branching instability is subsequently applied to a growing crystal, the result is called an ice dendrite [26] (Figure 3). The amount of branching is directly proportional to the atmospheric pressure, i.e., the lower the pressure, the fewer the branches that are generated [21,22].

**Figure 4:**
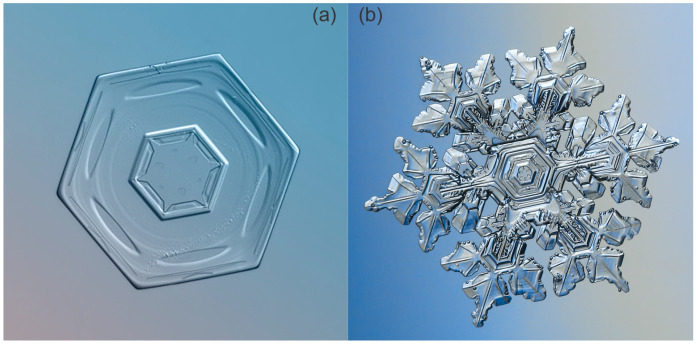
Faceted snowflake in the shape of a hexagonal prism (**a**) and branched snowflake with a complex structure (**b**) [29,30].

Crystal growth, however, depends on the balance between branching and faceting. The latter, in turn, tends to create flat crystal surfaces such as hexagonal prisms—the simplest shapes of snow crystals (Figure 4a)—while branching creates more complex structures (Figure 4b). Facets usually appear on many growing crystals because some surfaces tend to grow more slowly than others [17]. The interaction between these two processes depends on conditions such as temperature and humidity, so that crystals can grow with different characteristics [21,22].

Crystallography has become an important ally of biomimicry, since crystals are useful in various types of applications. Studying the physical and chemical properties of snowflakes has helped to understand how molecules condense to form crystals and how they grow [23]. The knowledge produced can lead to the proposal and future manufacturing of more efficient crystalline materials, as they are useful for the computing, electronics, and telecommunications industries [21,22].

The crystallography of snowflakes, beyond its practical application in materials, exemplifies how natural forms can influence both functionality and aesthetics. While advancements such as studies on snow particles [26] and fractal models of crystals have provided valuable insights, recent discoveries have deepened this potential. Works like those of Libbrecht [27], which examined the anisotropic attachment kinetics responsible for the formation of snowflakes’ characteristic hexagonal symmetries, and Sturm’s studies [20], which explored the fractal dimensions of these structures through stereoscopic microscopy, highlight promising geometric possibilities for bio-inspired architectural design.

Despite these advancements, gaps remain in the practical translation of these properties into material solutions, particularly in contexts requiring integrated thermal responsiveness. In this vein, the geometry of crystals, especially snowflakes, reveals untapped potential for constructive applications involving a complex structural organisation and lightweight forms. This potential could be further enhanced through the use of smart materials, such as thermo-responsive ones, paving the way for architectural solutions that are both aesthetically sophisticated and environmentally responsive.

In architecture, professionals can use the morphology of crystals to design the spaces, structures, or elements of a building. Transparency, for example, is another relevant factor that seeks to integrate environments, convey lightness, and allow natural light into spaces and is commonly associated with glass, acrylic, and other similar materials. In this sense, the use of translucent or transparent materials in projects contributes to a more fluid relationship between internal and external environments, promoting a sense of continuity and connection with the building’s surroundings.

### 1.3. Thermo-Responsivity

**Figure 5:**
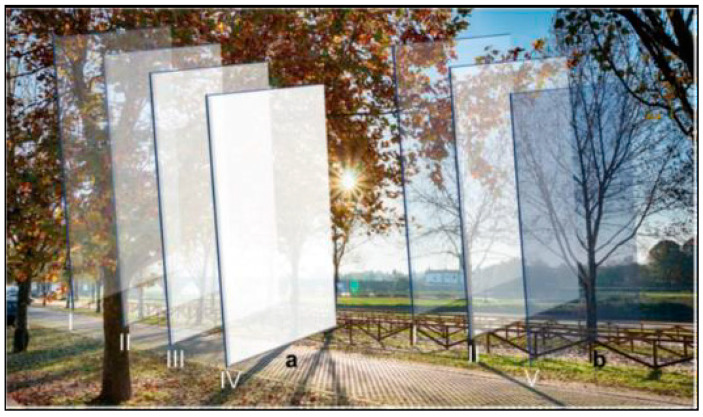
Schematic representation of an integrated system featuring an electrochromic device, which adjusts its transparency to become translucent (**a**), and a thermochromic device, which changes colour and transparency based on the solar radiation it receives (**b**) [31].

This term refers to the ability of certain materials or systems to alter their physical, chemical, or biological properties in response to temperature changes in structures that automatically adapt to environmental conditions. These thermo-responsive materials can alter their properties—such as colour, shape, or rigidity—enabling applications that include specific areas implemented in technological solutions, processes, or practices to meet particular energy and environmental needs (Figure 5).

According to Feng et al. [31], the materials represent an innovative solution to modern challenges, combining functionality and adaptability, as their characteristics allow them to modify their structure, shape, or behaviour in a reversible or irreversible way.

Thermo-responsiveness plays an important role in various fields of architecture. Materials with high thermal sensitivity adapt quickly to changes in temperature, offering almost immediate responses. This characteristic stands out for its versatility, being applicable in different sectors, both industrial and residential. It also contributes to energy efficiency by reducing dependence on active air conditioning and environmental control systems. Finally, thermo-responsiveness promotes sustainability, encouraging the conscious use of resources and minimising waste [31].

Within this framework, this research aims to discuss the integration of biomimicry, based on the study of snowflakes, and thermo-responsive materials for the development of an architectural pavilion. The pavilion, however, in this case, is merely a reference object that allows for the morphological and geometric study of a structure without restrictions regarding an architectural programme, as well as being a mobile structure that can be installed in different locations.

Despite numerous recent studies highlighting the use of thermochromic glass or hydrogels in façades and roofing systems for thermal regulation and the control or adjustment of systems to maintain desired operational levels, works that demonstrate the direct association between these materials and a bio-inspired construction system that seeks to reconcile formal lightness, transparency, and adaptability in a pavilion prototype were not found.

In this context, the primary issue this article aims to address is how the combination of geometries inspired by snow crystals (due to their lightness and crystalline order) and thermally responsive materials can overcome limitations observed in conventional pavilion structures, such as the lack of adaptability or high energy consumption.

This research seeks to integrate parametric modelling methods, algorithms for generating organic forms, and thermosensitive materials, resulting in a systematic approach to developing architectural structures that respond to environmental variations.

## 2. Methodology

This research adopts an interdisciplinary approach that integrates biomimetic principles, materials science, and architectural design. To this end, the methodology includes both a literature review and the execution of computational experiments.

Firstly, a comprehensive literature review was conducted, encompassing two main fields: classical references related to crystallography and biomimetics and more recent studies on thermochromic glass and hydrogels. This theoretical foundation was crucial for contextualising the use of advanced materials and integrating them into the framework of bio-inspired architectural design.

The morphological analogy with a snowflake, specifically a plate-type crystal, was chosen as the biomimetic reference due to its characteristics of lightness, transparency, and a crystalline organisation. Based on this analogy, a structural concept was developed that harmonised aesthetics and functionality, guiding the formulation of the architectural pavilion’s design. For this purpose, a problem-based approach was employed, which was subdivided into three phases: problem definition, exploration and research, and solution domain.

Subsequently, the selection and characterisation of materials were carried out, focusing on thermochromic glass and hydrogel, both recognised for their adaptive properties. Glass was adopted as the primary construction material due to its compatibility with these thermo-responsive materials. The characteristics of these materials, such as changes in their colour, rigidity, and thermal behaviour in response to temperature variations, were analysed based on data available in the literature.

In the experimental phase, computational resources, such as the Grasshopper software, were utilised to implement a parametric design approach. In this experimentation process, several restrictive parameters were defined, including the maximum area and height, to guide the development of the models. The purpose of the experiments was to investigate how bio-inspired shapes could be combined with thermo-responsive materials to create efficient, aesthetically appealing, and environmentally adaptive structures. In this context, the focus was on exploring architectural solutions that integrate high aesthetic and functional performance while also addressing sustainability and energy efficiency.

During the parametric modelling process in Grasshopper, a script was developed to generate isomorphic polysurfaces, which were subsequently rationalised into triangular glass panels. A mesh for the metal bar structure that supports the panels and forms the pavilion’s structural skeleton was also incorporated. This rationalisation was achieved using various tools within the parametric modelling workflow. Initially, six circumferences with different radii were positioned at various points in a three-dimensional space. Several Grasshopper-associated tools were utilised in the process: Galapagos facilitated the insertion and control of the circumferences’ radii; the Mesh Cocoon plug-in was then used to generate isomorphic polysurfaces based on the previously implemented circumferences; following this, the Kangaroo Physics plug-in triangulated the entire surface to create a mesh; and finally, the Weavebird plug-in enabled the subdivision of the mesh, forming the panels and the metal bar structure. This method facilitated the development of five design experiments inspired by the geometry of plate-type snowflakes, exploring bio-inspired shapes and patterns to maximise the aesthetic and functional performance of the structures.

As part of this study, two options for the application of thermo-responsive glass in the pavilion structure were proposed, considering different contexts of use. To evaluate the performance of these options, a comparative study of the materials was conducted, analysing criteria such as the type of glass, luminous transmission, solar factor, power consumption, solar heat gain, infrared radiation, material colour, and cooling index.

## 3. Results and Discussion

### 3.1. A Case Study—Reasoning Behind the Biomimetic Design of the Snowflake Pavilion

The objective of this study was to design a bio-inspired architectural pavilion that integrates thermo-responsive materials and explores the geometric and structural potential of biomimetic principles. The proposed pavilion aims to address challenges commonly faced in pavilion design, such as achieving a balance between structural efficiency, aesthetic appeal, adaptability to environmental conditions, and material sustainability. Traditional pavilion designs often struggle with optimising structural complexity, resource efficiency, and environmental responsiveness, which makes this biomimetic strategy particularly suitable for overcoming these limitations.

The proposal for a snowflake-based experiment used the biomimetic problem-based approach [32,33], also known as a challenge to biology [34], or even top-down [35] approaches, since there was a pre-established objective and only then was inspiration sought from nature. In this context, the problem can be described as the pursuit of ways to design an efficient and aesthetically innovative architectural structure that integrates thermo-responsive materials and adapts to environmental conditions. In this way, it was possible to analyse the snowflake from the perspective of morphological analogy [36], allowing for the interpretation of formal and structural attributes of the natural element.

The problem-based approach is divided into a few stages [32,33] that guide the process of abstraction and emulation of the bio-inspired element:

Problem definition: For the purpose of this research, the problem was simplified to clarify the limits of the proposal and direct the design process, the intention of which was to develop the geometry of an architectural pavilion. This simplification also helped to investigate references in nature that could inspire the project. Key-words were then established to define the desired characteristics of the pavilion: organic structure, use of few materials, transparency, and creation of a path inside.

**Figure 6:**
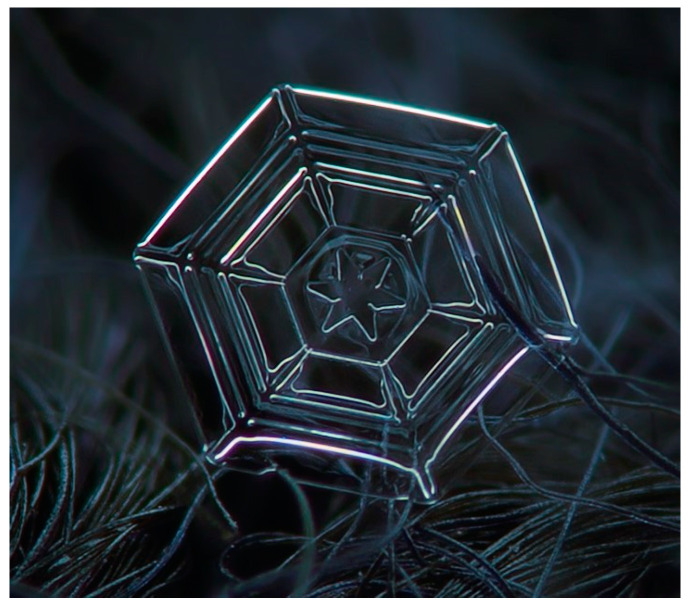
Plate-type crystal, forming a hexagonal prism [37].

Exploration and research: Snowflakes and their formation, types, and growth process were studied. Among the many types of flakes, it was decided to base this on a plate-type crystal (type P1 in the graphic in Figure 2). This type of crystal consists of a faceted hexagonal prism (Figure 6) which allows it to be rationalised into triangular shapes. The growth process of plate-type snowflakes originates from a central hexagonal core, with branches extending symmetrically as a direct response to specific environmental parameters, including temperature and humidity. These factors intricately govern the external morphology and also the internal structural arrangement of the crystal, resulting in a highly ordered and modular pattern of development.

**Figure 7:**
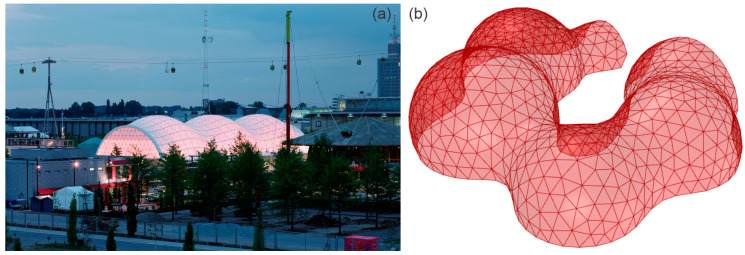
Isomorphic structure of the Japan Pavilion for the 2000 Hannover Expo, Germany, designed by Shigeru Ban Architects, featuring an innovative lattice structure of paper and wood [39] (**a**); parametric isomorphic mesh developed for one of the bio-inspired pavilion experiments (**b**).

Subsequently, a glass mesh was considered to surround the pavilion, and further research was carried out into a possible organic shape suitable for the experiment, and it was then determined that isomorphic polysurfaces (Figure 7) would be a relevant and appropriate solution from both a formal and functional perspective for the development of this pavilion proposal. From a formal perspective, isomorphic polysurfaces facilitate the generation of intricate, fluid geometries that align seamlessly with the organic and bio-inspired design objectives of this study. Functionally, their parametric construction affords a high degree of flexibility in optimising the equilibrium between structural efficiency and spatial adaptability, ensuring a responsive and contextually integrated solution. These polysurfaces are amorphous objects constructed as sets composed of parametric objects with internal forces of mass and attraction. They exert fields of influence that can be additive (positive) or subtractive (negative). This type of geometry is formed by computing a surface on which the composite field has the same intensity. In the realm of bio-inspiration, the aggregation and growth of ice crystals, driven by molecular forces and shaped by environmental conditions, give rise to highly ordered geometries characterised by symmetric and repetitive patterns. Similarly, the computational modelling of isomorphic polysurfaces utilises fields of influence to define their resultant geometry, embodying a delicate equilibrium of forces that reflects the structured yet inherently adaptable nature observed in snowflake formation. In general, the surface boundary of the whole snowflake moves as the influence fields change their location and intensity [38]. This approach enables the exploration of complex and continuous shapes that can adapt to the restrictive parameters and spatial requirements of the project (Table 1), ensuring a seamless relationship between form, structure, and function.

The pavilion’s structural composition was inspired by the geodesic association that segments arcs into bars, which would allow the desired isomorphic polysurfaces to be triangulated. However, the structure would not originate from polyhedra and would not be based on regular solids. Furthermore, the bars would not all be the same size and would not form equilateral triangles, so the structure would not fit into a geodesic idea but would only be used as inspiration [40].

Solution domain: In this last phase of the problem-based approach, the analogy is contextualised, the biomimetic principles are applied, and logical knowledge is transferred to the practice of the experiment. In this step, a connection is established between the idea and the technology, bringing the pavilion to reality through simulations and tests [33].

This stage is crucial for transforming conceptual ideas into tangible solutions, exploring how biomimetic principles can be translated into practical outcomes that meet the project’s technical, functional, and aesthetic requirements. Furthermore, it facilitates constant iteration between solutions and adjustments, ensuring a coherent integration of biomimetic concepts and computational tools into the architectural design. In this specific context, this phase played a key role in incorporating the morphological analogy of the snowflake into the pavilion’s design while simultaneously integrating the selected thermo-responsive materials with the bio-inspired geometry.

**Figure 8:**
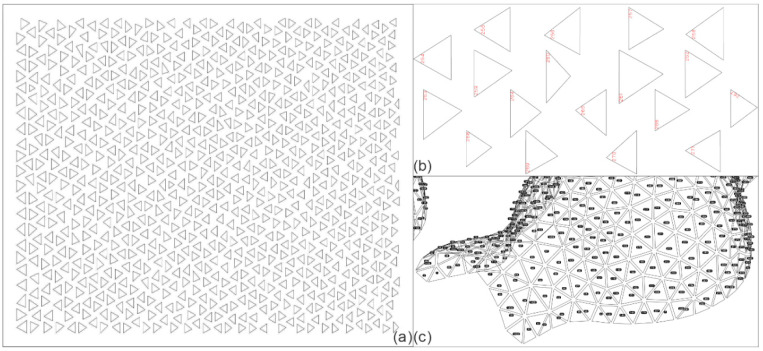
Mapping of some panels of the pavilion (**a**); enlargement of some panels with their respective identifications (**b**); location map of each panel using its identification number (**c**).

The biomimicry of the pavilion: After going through all the logic proposed by Badarnah [33] for this experiment, it is possible to point out important characteristics that relate to the natural inspiration—a snowflake. The hexagonal symmetry of a plate-type crystal was divided into 6 triangles and applied in a mesh based on 6 spheres. The nature of the different panels goes back to the uniqueness of snowflakes, considering that each panel has its own measurement and identity; for this, the RhinoNest tool was used within the Rhinoceros modelling software, which allowed for the pieces to be mapped (Figure 8). As far as the metal structure is concerned, it supports the panels and forms the pavilion just as branches provide structure to snowflakes.

Finally, with the script already implemented, it was possible to adjust parameters so that the pavilion would remain within the standards of previously established limits, such as a maximum area of 800 m^2^ and a maximum height of 8 m, as shown in Table 1. To leverage the full potential of parametric modelling, a morphological study was also carried out for various options for pavilions with the same characteristics while modifying only certain parameters described in the following section.

### 3.2. Case Study—Snowflake Pavilion Experiments

**Figure 9:**
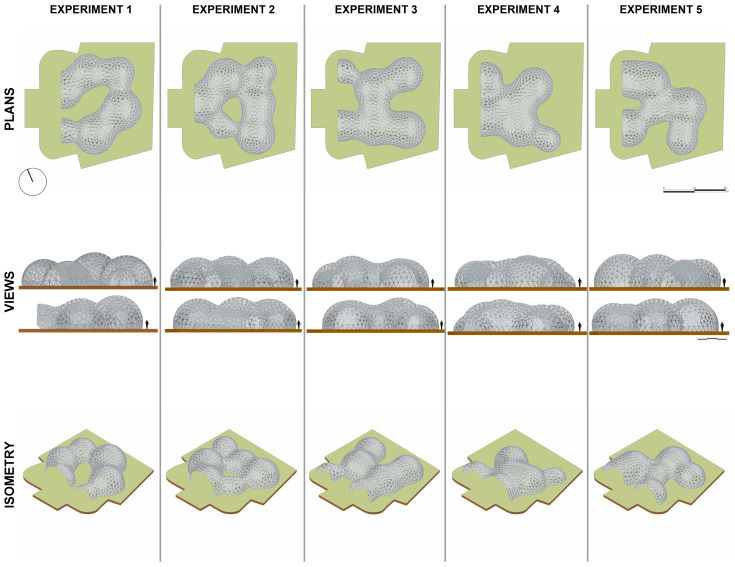
Schematic documentation of the five experiments.

The bio-inspired architectural pavilion project was made up of five experiments developed in Grasshopper as a parametric computer environment (Figure 9 and Table 1). However, these experiments have only been recorded as a presentation of the diversity of the results that can be achieved, when in fact countless proposals can be developed from the different possible combinations of implemented parameters.

The use of Grasshopper and the aforementioned plug-ins is justified by the possibility of carrying out complex parametric modelling, which allows for different formal and functional solutions to be explored efficiently. In addition, Grasshopper enables integration with other design and simulation tools, optimising the workflow and ensuring greater precision in the results. Plug-ins complement this functionality by offering more specific features, such as environmental analyses, structural simulations, and the generation of optimised shapes, which extend the software’s capabilities and make the design process more robust.

It is noted that morphological variability still depends on the location of the insertion points (x, y, and z coordinates) of each sphere. These points actually represent the position where the centre of the sphere is inserted into three-dimensional space (Table A1). This dependence reinforces the direct influence of the spatial configuration of these points on the definition of the shapes generated, since the interaction between the spheres occurs based on their relative positions and the extent of their radii.

In this sense, the precise distribution of the x, y, and z coordinates is important in determining both the geometric pattern and the structural variability of the configuration. In addition, changes in these points can result in significant variations in the connectivity, density, and uniformity of the shapes produced. This shows that choosing and controlling these coordinates is not just a technical step but also a design element that can be exploited to achieve specific results in the design context.

**Figure 10:**
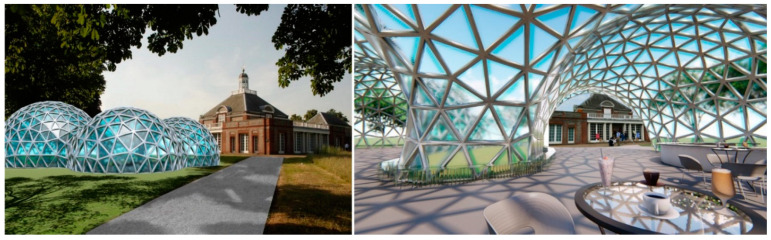
External and internal perspectives of the pavilion from experiment 1.

In this regard, some restrictive parameters were implemented. These were a maximum height of 8 metres, a maximum radius of each sphere of 25 metres, and a maximum area of 900 m^2^. These measures were set arbitrarily so that the pavilion could be used for presentations, public spaces, cafés, fairs, etc. (Figure 10). The number of spheres was not considered a restrictive parameter; however, all experiments were conducted based on the insertion of 6 spheres.

**Figure 11:**
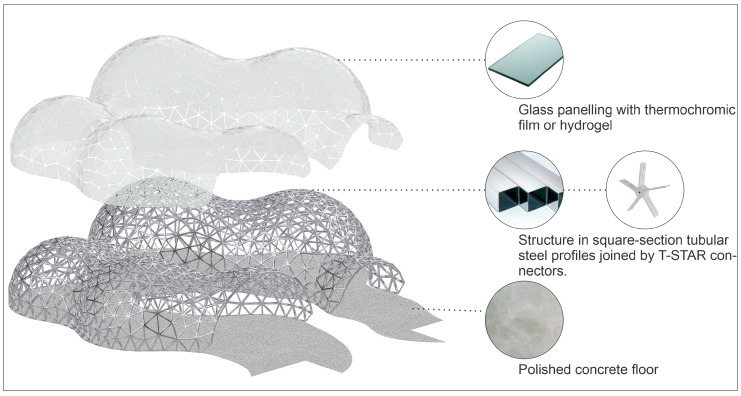
Exploded perspective of experiment 1.

In terms of materials (Figure 11), the pavilion consists of a metal structure made of square tubular profiles with a square-section of 50 × 50 mm. This structure incorporates a robust system of T-STAR^i^ connectors in a star shape, joined by flat steel bars that are welded and bolted together. The roof is composed of laminated glass panels with a thermochromic film or hydrogel overlay, adhered to the metal structure using VHB^ii^ tape. The flooring would employ polished concrete techniques for a durable and refined finish.

Within these experiments, it was determined that the smaller the size of the cells (glass panels), the more uniform the surface becomes and the lower the level of triangulation, the fewer the faces that are generated while the higher the level of triangulation, the more the faces that are generated in the mesh. Only the restrictive parameters determine a limitation, the others (number of spheres, cell size, level of triangulation, dimensioning of the metal structure, thickness of the glass panels) are subject to adjustments according to the appropriate structural dimensioning. No model took into account precise structural dimensioning.

This geometric behaviour becomes relevant when evaluated in scenarios that value sustainability and material efficiency. The possibility of adjusting the size of the cells to achieve greater uniformity makes it possible to improve aesthetics and functionality, as well as potentially reducing waste and optimising resources. While detailed structural calculations have not been conducted at this stage, the notion of efficiency discussed here is conceptual, deriving from the intrinsic benefits of geometric uniformity. By minimising irregularities and improving spatial organisation, such uniformity has the potential to address inefficiencies typically linked to uneven load distributions or material redundancies. In addition, geometric uniformity can optimise structural performance, allowing for a better load distribution and reducing the material needed to support a structure [41,42]. In architectural applications, this approach can contribute to the better management of natural light, greater thermal comfort, and a more efficient use of metal structures and glass, aligning with sustainable design principles that seek to align functional efficiency and human well-being.

## 4. Thermo-Responsive Envelope

### 4.1. Thermochromism

Thermochromism is the property of certain materials to change colour in response to variations in temperature. This occurs due to changes in the molecular structure of the material or in the way the molecules interact with light as the temperature increases or decreases. Thermochromic materials can be liquids, solids, polymers, or even liquid crystals.

In the case of advanced materials and high-tech applications, thermochromism also appears in fields such as architecture and construction, where thermochromic glass and coatings can be used for passive temperature control in buildings. These materials become lighter or darker as the room temperature varies, helping to reduce the need for artificial heating or cooling, which results in energy savings and thermal comfort [43].

Solutions can be achieved by selecting construction methods and materials that promote thermal insulation. With regard to glazed areas present in modern façades, it is important to consider the layout of the spaces, as well as their exposure to and protection from sunlight.

Researchers such as Tavil et al. [44] provide a detailed exploration of the characteristics and advantages of thermochromic glass, emphasising its ability to regulate light transmission based on ambient temperature. This enables the control of heat and natural light ingress. They note that the material’s effectiveness varies according to climatic conditions and building orientation. In hot climates, thermochromic glass helps reduce cooling loads, while in temperate regions, it aids in balancing solar heat gain. Similarly, Feng et al. [31] assert that the opacity of thermochromic glass changes due to thermochromic materials, which block more light at higher temperatures, enhancing thermal comfort and energy efficiency in buildings.

Additionally, Salamati et al. [45] and Zhao et al. [46] highlight that inorganic materials such as vanadium oxide are more commonly used in construction due to their durability and thermal modulation capacity, whereas organic materials are more prevalent in temporary or low-cost applications. These authors further emphasise that thermochromic glass does not require sensors or control devices, as its changes occur naturally within the material, promoting a sustainable approach and reducing reliance on mechanical systems.

Some drawbacks in architectural applications have also been identified by the aforementioned authors. Thermochromic glass is more expensive than standard glass, and its efficiency can be limited in very cold or hot climates, where additional thermal control measures are still required. Furthermore, its thermochromic behaviour is directly dependent on ambient temperature, preventing manual or customised control. The aesthetic aspect, with the glass changing colour, may not suit all designs and can potentially reduce visibility in its darkest state. Lastly, installation can be more complex, requiring specialised knowledge, which increases both project costs and the execution time.

**Figure 12:**
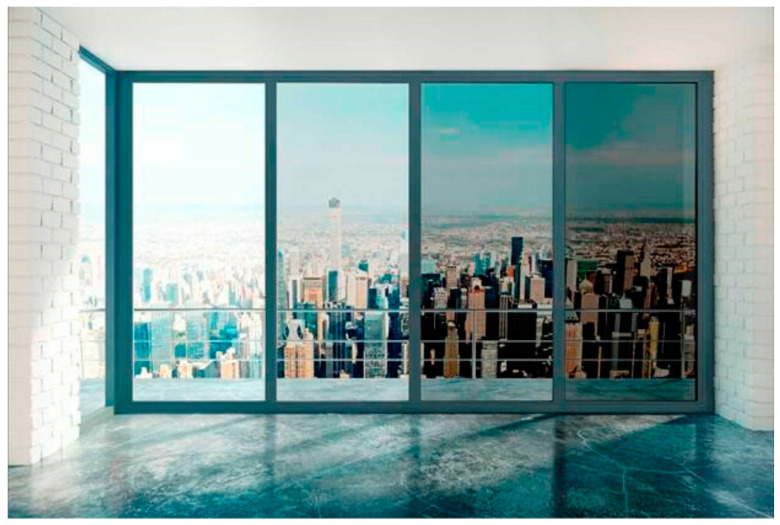
Thermochromic glass with property changes based on solar heat [48].

The applicability to glass represents an innovative technology that interacts naturally with sunlight, changing its colour in response to heat exposure. As UV^iii^ ray incidence increases, the glass darkens. According to Chohfi [47], the uniqueness of this material lies in its ability to change colour without the need for electricity or human intervention, functioning as a completely autonomous and mechanical process (Figure 12).

Thermochromism is produced by applying a special film of vanadium oxide (VO2) to the surface of the glass. The basic elements and reactions to the transparent colour tones correspond to the increase in temperature due to the effects of solar radiation. When infrared rays hit the glass, the vanadium film absorbs the heat and darkens as the temperature rises.

**Figure 13:**
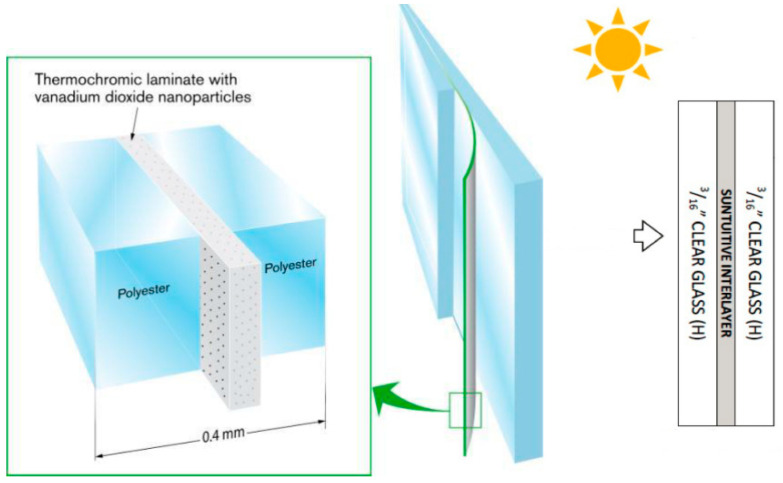
Construction principles for lamination foil incorporating thermochromic nanoparticles, along with the composition of glasses and films [49].

The composition of the glass, which includes lamination and the use of PVB^iv^ (polyvinyl butyral) films, also provides partial barriers against heat penetration. The amount of ambient lighting will also depend on the glass cores used. This film can be applied to nearly all types of laminated and tempered glass (Figure 13) and converted into thermochromic glass, which is one of the glass industry’s most promising innovations for breaking paradigms in civil construction.

The integration of these specialised glass types would help enhance overall performance and address existing limitations, making thermochromism even more efficient. These glass technologies are gaining popularity among models with similar characteristics.

Although still relatively new, some international glass industry companies have already invested in their production, particularly in Central and Northern Europe, Japan, the United States, and Canada. These regions produce or utilise thermochromic glass primarily in sustainable construction and technological innovation projects.

These characteristics make thermochromic glass an exceptionally effective option for architectural projects aiming to combine comfort, energy efficiency, and aesthetic appeal. Its ability to adapt to temperature variations and its positive impact on sustainability are positioning it as an essential technology in the construction industry.

### 4.2. Hydrogels

Hydrogels are materials made up of highly hydrated polymer networks capable of changing their physical properties (opacity and colour) in response to external stimuli such as temperature, light, or humidity.

**Figure 14:**
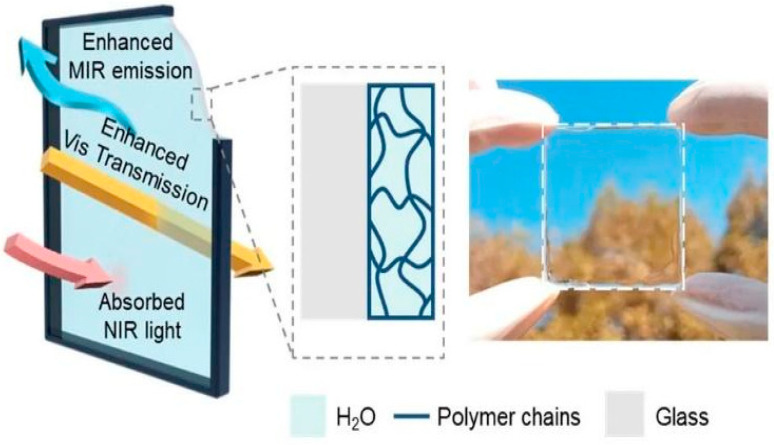
Diagram illustrating the properties of hydrogel glass [51].

Scientists at Wuhan University in China [49] have created an innovative smart glass based on a hydrogel, which allows more light to pass through and reduces heat ingress. With a thickness ranging from just 0.3 mm to 0.7 mm, this material reflects infrared rays from the outside, allowing part of the light spectrum to penetrate the internal environment (Figure 14).

According to the researchers, glass windows have been developed with a new design and are designed to admit visible light and illuminate the interior of buildings. However, interactions with infrared radiation, perceived as heat, contribute to increased energy consumption, especially during the summer months. These windows help keep indoor spaces cooler, offering environmental benefits and reducing construction costs [50].

According to Sun et al. [50], laboratory experiments revealed that glass manufactured from HBPEC^v^/PNIPAM^vi^ hydrogel is more suitable and demonstrates higher light transmittance, solar modulation, and internal temperature regulation compared to traditional glass. This would help maintain a more comfortable indoor environment, similar to other radiative cooling systems, without increasing energy consumption.

The researchers also noted that hydrogels are easy to produce and offer a cost-effective alternative, making them relatively simple to implement. This accessibility gives them an advantage over more complex solutions.

### 4.3. Comparative Analysis

Based on the two materials presented earlier, it was observed that these technologies offer significant sustainable benefits. This can be seen in the thermochromic glass composition model, which represents a developing solution but has not yet been widely adopted in urban buildings. Although there is a considerable lack of awareness about this technology, research on thermochromism has achieved advancements in energy efficiency. In the adaptation of the hydrogel experiment, the cooling index stood out as a key feature, making it a strategic choice for reducing heat entry while regulating light transmission into interior spaces. Table 2 summarises the properties of the glasses studied, providing better specifications for the case study.

According to an assessment of Table 2, it is evident that both types of glass demonstrated a balanced performance, showcasing different strategies in their properties. In Glass 1, two transparent glass panes are used with an intermediate layer of thermochromic PVB. On the other hand, Glass 2 utilises a single monolithic clear glass pane combined with a hydrogel-based material (a chemical structure that enables cross-linking between polymer molecules and layers of water).

The hydrogel glass proved to be highly effective, offering better solar protection than thermochromic glass. Conversely, the latter allowed for greater light entry, indicating better light transmission due to its colour variation in response to UV ray incidence, ranging from high to low.

In terms of temperature control within the environment, hydrogels demonstrate superior heat absorption or reflectance, owing to the hydrogel’s composition, which can also regulate humidity. Meanwhile, thermochromic glass reduces heat transmission by darkening at high temperatures.

This table indicates that hydrogel glass does not need to adopt a darker colour than conventional glass to maintain thermal effectiveness. By varying the thickness of the hydrogel layer, up to 92.8% of visible light can pass through, compared to 92.3% for conventional glass.

In summary, hydrogels offer a more versatile and responsive approach, adapting to various stimuli (temperature and humidity) and being suitable for environments requiring enhanced thermal comfort control and multifunctional properties. Regarding costs, hydrogels can range from moderate to high, depending on the hydrogel’s composition and application, facilitating its implementation and giving it an advantage over thermochromic glass, where its glazing application option becomes higher due to the coating technology.

## 5. Conclusions

The integration of biomimetics, architecture, and thermo-responsive materials reaffirms the potential of this interdisciplinary approach to developing sustainable and adaptable architectural solutions. This study explored how the geometry of snowflakes, specifically, plate-type crystals, can inspire the creation of lightweight, thermo-responsive, and transparent structures—key characteristics that shaped the conceptual framework of the proposed pavilion.

Designed with adaptability and efficiency as guiding principles, the pavilion served as an exploratory model to demonstrate how thermo-responsive materials can be utilised to provide thermal comfort across diverse climatic contexts. It is important to emphasise that the discussion on thermal comfort was grounded in a review of the literature and theoretical assumptions regarding the behaviour of thermo-responsive materials. This therefore points to the possibility of future studies, such as computational simulations, to analyse airflow and thermal performance, as well as experimental validations of material properties, including the thermochromic response of glass and the heat absorption and release capacity of hydrogels under the proposed design conditions.

Thermochromic glass proved particularly versatile, performing well in both hot and cold climates, while hydrogel showed promise in mitigating overheating in warmer environments. While these observations are primarily based on general material performance studies, their conceptual application to the pavilion highlights their potential to address the specific challenges posed by varying environmental conditions. This reinforces the idea that site-specific characteristics and project requirements must guide material selection to achieve optimal results.

The use of a parametric design, facilitated by Grasshopper, enabled the investigation of geometric configurations inspired by snowflake morphology. The creation of isomorphic surfaces and their rationalisation into triangular panels effectively integrated form and function, achieving a balance between visual coherence and the conceptual potential for optimised structural behaviour.

This study underscores the importance of interdisciplinarity for architectural innovation, bridging materials science and biomimetics. Although this research focused on the conceptual feasibility of the pavilion rather than its implementation, it highlighted the technical and aesthetic potential of thermo-responsive materials within biomimetic design frameworks. Future research should prioritise practical validations in real-world contexts, assess economic feasibility, develop and/or apply more accessible materials, integrate automation technologies, and facilitate the expansion of interdisciplinary collaborations to optimise biomimetic solutions.

In summary, the pavilion serves as a laboratory of ideas, illustrating how biomimetics, coupled with advanced design tools, can inspire innovative architectural approaches. While exploratory in nature, this study lays the groundwork for future investigations into adaptive, sustainable designs that merge aesthetics with environmental responsiveness.

## 6. Notes

^i^ T-star connectors (T is for tubular) are star-shaped structural components used to facilitate the assembly of tubular metal profiles by providing multi-directional connections, ensuring structural stability and ease of assembly. They are commonly fabricated from steel and are designed to accommodate high loads and resist mechanical stresses.

^ii^ VHB (Very High Bond) is a high-performance adhesive tape by 3M, designed for the durable bonding of materials such as metal, glass, and plastics, offering strong adhesion and resistance to weathering and thermal expansion.

^iii^ UV (ultraviolet) rays are a type of electromagnetic radiation emitted by the sun.

^iv^ PVB (polyvinyl butyral) is a widely used thermoplastic polymer known for its unique properties, such as transparency, adhesion, flexibility, and impact resistance. It is commonly utilised in the laminated glass industry, particularly in applications requiring safety and durability.

^v^ The HBPEC hydrogel (Biomimetic Hydrogel of Cross-linked Polymers) is a biomimetic technology developed for various medical and industrial applications. It is typically composed of biocompatible polymers chemically cross-linked to form a three-dimensional network capable of retaining large amounts of water or fluids.

^vi^ PNIPAM hydrogel (Poly(N-isopropylacrylamide)) is a thermo-responsive polymeric material widely studied for its ability to respond to temperature changes. Its key feature is a phase transition at a specific temperature known as the “Lower Critical Solution Temperature (LCST)”, which typically occurs at around 32–34 °C in water.

## Figures and Tables

**Table 1:** Summary of the parameters used in each experiment.

	Experiment 1	Experiment 2	Experiment 3	Experiment 4	Experiment 5
Area (m^2^)	712.17	818.20	740.17	663.17	682.10
Maximum height (m)	7.10	7.04	6.92	6.66	6.88
Number of spheres	6	6	6	6	6
Minimum radius (m)	15.12	15.12	15.12	15.12	15.12
Maximum radius (m)	24.83	24.77	24.83	24.83	24.83
Cell size	2	2	2	2	2
Level of triangulation	3	3	3	3	3
Dimension of the steel profile (cm)	5 × 5	5 × 5	5 × 5	5 × 5	5 × 5
Thickness of the glass panels (mm)	8	8	8	8	8
Quantity of panels	1468	1669	1714	1250	1448

**Table 2:** Summary of properties of the glasses analysed [47,50].

Properties	Thermochromic Glass (Glass 1)	Thermochromic Hydrogel HBPEC/PNIPAM (Glass 2)
Type of glass	Colourless laminated	Colourless monolithic
Luminous transmission	60–80%	87.5%
Solar factor	40–70%	71.2%
Power consumption	20–43%	44.6%
Solar heat gain coefficient (SHGC)	18–34 °C	24.1–33.2 °C
Infrared radiation (IR)	50–92.3%	70–92.8%
Material colour	Light to dark	Opaque
Cooling index	Solar variation	3.5 °C

## Data Availability

Data are contained within the article.

## Appendix A

**Table A1:** Location of the centre of each sphere implemented in each experiment.

Experiments	Points (ID of the Spheres)	Point X (Location of the Centre)	Point Y (Location of the Centre)	Point Z (Location of the Centre)
1	0	23.646086	2.246508	2.284453
	1	41.732537	48.035315	1.692167
	2	1.029938	34.918252	0.07004
	3	43.931842	20.4339	3.978003
	4	0.30941	4.954323	0.45411
	5	19.160484	48.747392	2.19186
2	0	4.252415	25.601162	3.807322
	1	49.177834	49.554285	3.671784
	2	44.204777	2.353636	3.081378
	3	22.145973	44.026648	2.781297
	4	18.849994	8.433083	1.29413
	5	46.478513	25.948052	0.702627
3	0	25.888618	31.588639	3.419466
	1	1.754111	4.051676	3.967149
	2	49.914747	0.983414	2.432717
	3	26.236869	3.973899	1.989577
	4	3.126016	44.661695	2.297498
	5	49.301309	41.959278	1.470245
4	0	28.131149	10.90231	1.264213
	1	3.43477	47.127327	2.821347
	2	38.628121	40.996392	0.679764
	3	0.946566	18.552405	2.700611
	4	49.087683	1.343544	0.01573
	5	22.838666	28.157425	2.637039
5	0	19.161023	43.532784	3.304693
	1	43.333693	1.251298	3.735008
	2	3.870861	9.494546	1.184934
	3	43.556857	24.656106	3.171806
	4	22.99581	21.419026	3.40676
	5	2.010796	46.153246	2.651866
